# Natural hazards and climate change are not drivers of disasters

**DOI:** 10.1007/s11069-021-05100-1

**Published:** 2021-11-10

**Authors:** Alik Ismail-Zadeh

**Affiliations:** 1 Karlsruhe Institute of Technology, Applied Geosciences, Karlsruhe, Germany; 2 Institute of Earthquake Prediction Theory and Mathematical Geophysics, Russian Academy of Sciences, Moscow, Russia

**Keywords:** Disaster, Vulnerability, Exposure, Natural hazard, Risk, Climate change, Preparedness, Public awareness

## Abstract

Many nations face challenges in assessing, understanding, and responding to the time-dependent nature of disaster risk. Changes in the intensity of occurrences of extreme events coupled with changes in vulnerability and exposure alter the impacts of natural hazards on society in mostly negative ways. Here an interrelationship between natural hazard (NH), climate change (CC), vulnerability (V), exposure (E), and decisionmaking (DM) is considered. While NHs trigger disasters and CC is likely to intensify occurrences of disasters, V and E present major drivers of disasters. Informed DM on disaster risk reduction should be based on scientific evidence from NH and CC, knowledge of V and E, and relevant options for actions on preventive disaster measures as a part of preparedness and public awareness.

## Introduction

On 1 November 1755, when Roman Catholics observe All Saints Day, people went to churches in Lisbon (Portugal) to pray for the Almighty God. Nobody expected that this day will enter the history of the mankind as a day of one of the most destructive disasters in Europe. A great earthquake ruptured the Earth’s crust in the Atlantic ocean and generated seismic waves (Gupta and Vineet 2013), which traveled to Lisbon causing a widespread destruction of buildings and heavy casualties. Those, who could escape from the crushing churches and houses, run toward the ocean to take boats and leave the city. But they met great tsunami waves generated by the earthquake, which swept them into the ocean.

The disaster happened at the time called the Age of Enlightenment, when scholars and philosophers believed that the God created “the best of all possible worlds” (Leibniz 1985). After the Lisbon disaster, Voltaire, a French philosopher, asked: “Is this the best of all possible worlds?”, and answered: “And if so, how would then the others look like?” (Wade 1959). Despite the notion of risk had already been developed in Europe, which assumed that the future depends on human decisions rather than on providence, still most of the population, who suffered due to the disaster, believed that “the God arranged the things that happen to us” (Fuchs 2009).

Meanwhile, in a year after the Lisbon disaster, Immanuel Kant, a German philosopher, wrote a book about the earthquake and its consequences, where he highlighted the social and engineering backgrounds of disasters rather than its natural triggering mechanism. He wrote: “If humans are building on inflammable material, over a short time the whole splendor of their edifices will be falling down by shaking. However, is this reason to blame providence for it?” (Kant 1756). Despite that the knowledge of two basic drivers of disasters—vulnerability (V) and exposure (E)—started to emerge in the eighteenth century, the deep and clear understanding of the drivers was still in its immature form.

With the termination of the Cold War and the advent of perestroika,^1^ which began in the Soviet Union in 1980–1990s, the dreadful scenarios of nuclear catastrophe began to be forgotten, and the world breathed a sigh of relief. With this, the concepts of catastrophes and disasters moved from the sphere of real threats to the sphere of political declarations, which led many governments to treat the problem of risks with a degree of relative calm. At the same time, disasters of the early twenty-first century (e.g., the 2003 heat waves in Europe; the 2004 Indian Ocean and 2011 Great East Japan earthquakes and tsunamis; the 2005 Hurricane Katrina and 2012 Hurricane Sandy; the 2010 Haiti earthquake followed by a cholera outbreak, among other catastrophes caused by natural hazard (NH) events) began to alarm societies and governments to take preventive measures to save people, their property, and infrastructure. However, the physical and social vulnerabilities of our civilization to natural and human-induced hazards are still growing due to the increase in the number of vulnerable objects and clustering of populations and infrastructure in the areas prone to NHs (Ismail-Zadeh 2018a). Particularly, using satellite imaging, Tellman et al. (2021) show that the total population in locations with satellite-observed inundation grew by 58–86 million from 2000 to 2015, and accounting for climate change (CC) projections for 2030, the proportion of the population exposed to floods will increase further.

Despite advances in NH understanding, monitoring and forecasting, disaster losses continue to raise at local to global scales, and the NH impacts become more taxing and hence constraining states in their abilities to undertake the losses without external assistance. It is not necessary a natural hazard event to be extreme to cause extreme impacts and damages. Consider the 2010 Chile magnitude 8.8 earthquake and the 2010 Haiti magnitude 7.0 earthquake. The energy release during the Chile earthquake was greater than that during the Haiti earthquake by a factor of about 390, while the death toll ratio was about 0.0022 presenting an inverse proportionality with respect to the earthquake magnitude ratio, namely, about 550 victims in Chile versus more than 250,000 victims in Haiti.

NH events cause often severe impacts across national borders. Namely, earthquakes and tsunamis (e.g., the 2011 Great East Japan disaster resulting in a nuclear incident and disruptions of supply chains affecting many countries), volcanoes (e.g., the 2010 Eyjafjallajökull volcanic eruption leading to a large airline traffic shutdown in Europe), wildfires (e.g., the 2021 wildfires in Southern Europe), floods (e.g., 2021 flooding in Western Europe), or viruses (e.g., Ebola or SARS) can impact several states and even become a global disaster (e.g., the COVID-19 pandemic). Impacts of NH events highlight the interconnectedness of global society, even thought the events triggered extreme impacts have normally local origins (Ismail-Zadeh and Cutter 2015; Ismail-Zadeh 2020a).

## Vulnerability and exposure are main disaster drivers

Vulnerability and exposure to NHs have been considered as key determinants of disaster risk and the main drivers of disaster losses (e.g., Cardona et al. 2012). Significant knowledge about dynamic features of V and E has been accumulated for the last decades, and various approaches to their assessments have been developed (e.g., Cutter et al. 2003; Wisner et al. 2004; Lavell et al. 2012; Birkmann et al. 2013; Birkmann 2014; Ismail-Zadeh et al. 2014; Fuchs and Thaler 2018). Particularly, Birkmann et al. (2013) provided a background to framing V in the NH context and CC adaptation, and developed a holistic framework for V assessments, exposed values at risk, and the adaptation. Social processes responsible for V and exposed assets at risk, such as, unsustainable development, increasing urbanization, social inequalities, and livelihood disparities, can amplify the NH impacts and drive increased losses, especially in coastal and riverine regions (Ismail-Zadeh and Cutter 2015).

NHs and CC are quite often considered by natural scientists and policymakers as the principal drivers determining the severity of “natural” disasters. For the last several decades, the social science community dealing with disasters and risks put tremendous efforts to explain that disasters triggered by natural events are not “natural” but social phenomena, and that V and E emphasize the social construction of risk (e.g., Pelling 2001; Birkmann et al. 2013; Mizutori 2020). Representatives of 195 States, who signed the Sendai Framework Agreement on Disaster Risk Reduction 2015–2030, agreed that disasters that progress from NHs are not “natural”, and risk reduction should be the primary focus in disaster mitigation and prevention. However, today we hear that CC is behind the floods in Germany and neighboring countries (e.g., Cornwall 2021). Indeed, the CC contributes to the increased frequency and severity of hydro-meteorological extreme events (IPCC 2012, 2021), and its impacts will increase burdens on the populations, which are already vulnerable to natural hazards (Adger 2006). However, the CC is not a driver of disasters, but is very likely to contribute to the frequency of future disasters, if no preventive measures related to its anthropogenic component are considered.

Gilbert White wrote that "floods are 'acts of God' but flood losses are largely acts of man" (White 1945). But why still some natural scientists see disasters caused by NHs as “natural” events? Natural scientists investigate physical, biological, chemical, and other natural phenomena that may lead to hazard events, considering that better understanding of extreme NHs would result in disaster risk reduction (DRR). Moreover, natural scientists typically do not deal with social V (as social scientists) or physical V (as engineers) or exposed assets (as insurance and re-insurance industries) to assess risk as a convolution (an integration over space and time) of NH, V, and E. And hence, understanding of the dynamic interaction between NHs, V, E, and CC is critical and should be improved.

NHs may trigger disasters, but they do not drive disasters. A strong earthquake, severe storm or flash flood occurring far from a populated region will not typically generate a disaster. The 2010 devastating earthquake of magnitude 7.0 occurred in the close vicinity to Port-au-Prince, the capital city of Haiti, took lives of more than a quarter of million. Meanwhile, the 2021 magnitude 7.2 earthquake occurred along the same major fault zone in Haiti at a distance of about 100 km from the 2010 earthquake epicenter generated much less losses (about 2200 casualties, as on 22.08.2021), because the 2021 earthquake’s epicenter was located not in the highly populated region. Virus SARS-CoV-2 (a biological hazard) would have not turned to become the COVID-19 disaster, if the society has been less vulnerable and better prepared, i.e., if the public had been aware how to live with the risk and how to act in the case of emergency (Ismail-Zadeh 2020a).

## Preparedness and awareness

Although the knowledge about local V and E are normally available for policymakers, unpreparedness of states and public unawareness lead in many cases to local, national, regional, and global disasters (Ismail-Zadeh and Takeuchi 2007; Ismail-Zadeh 2020a, 2021). Preparedness and awareness are important factors in disaster risk mitigation and help to ensure that people can act appropriately on warnings issued. Preparedness and awareness assume that (1) sound scientific and practical information and resources are available to people; (2) the psychological and social capital and capacity are available for interpretation and local use of the information and resources; and (3) responsibilities of civil agencies and communities in disaster risk management are shared (Ismail-Zadeh and Cutter 2015).

The level of public awareness of risks associated with flooding as well as the preparedness for flood events proved to be extremely low during the 2021 flash flooding events in Germany, partly because they happened without a warning. “But even if a warning had been sent to the appropriate local authorities, it is unlikely that it would have been delivered to the public … in a timely manner”, as Ismail-Zadeh et al. (2017) mentioned already with respect to the 2004 Indian Ocean earthquake and tsunamis. When Germany decided to test its national warning system in 2020 to raise public awareness about potential emergency events, it was revealed that the test was unsuccessful as “sirens did not go off in many places across the country—because they’d been dismantled after the end of the Cold War—and there were delays in the message getting through on Germany’s warning smartphone apps” (Loxton 2021). “It is also unlikely that people would have responded to it in appropriate manner” (Ismail-Zadeh and Takeuchi 2007), since most of the local community did not know that flash floods can occur near their houses and did not believe that such a disaster, as happened in July 2021, could affect them. In addition, local people did not know where to escape in the case of floods or other (e.g., severe storms or wildfires) emergency, and how to protect their lives and properties. Public education in DRR is an essential component in raising awareness of the potential risks of extreme events and in enhancing public safety, and it constitutes a prerequisite for effective risk management strategies (e.g., Ismail-Zadeh and Takeuchi 2007; OECD 2010).

In Japan, the preparedness to NHs and awareness of people regarding NHs are the highest in the world. Residential houses and other building constructions are resistant to strong earthquake or severe storms, sea walls and levees protect people against tsunamis and floods. Local people are well aware about the risk they live with, and they know how and where to evacuate after an alert issued. Natural scientists from Japanese universities and academic institutions organize workshops for local people to present their sophisticated models, which realistically describe consequences of large earthquakes, tsunamis, floods, storms, and other NH events. Together with local authorities and social scientists they explain how people should act and protect their lives and property in the case of extreme events. Disaster management during the 2021 eruptions of La Palma volcano and lava flows on the Canary island showed an example of good practice in preparedness, early warning, and evacuation, despite 50 years passed since the last strong eruption on the island. These important lessons are still to be learned by European and other states.

Preventive measures are not a panacea for disasters, but in most cases, work well enough to save lives and properties. For example, the underestimation of the height of tsunami waves and potential inundation immediately after the 2011 Great East Japan earthquake led some people to do not evacuate their building to safer places after alerts were issued. They believed that available engineering constructions would protect their houses and lives. Unfortunately, the tsunami waves were much higher than expected, and seawalls were incapable to prevent the great inundation (Ismail-Zadeh 2018b). On the other hand, a frequent warning issued with overestimated heights of tsunami waves plays a negative role, as the coastal community begins to disbelieve tsunami alarms (Ando et al. 2011). The same is relevant to the people living on banks of rivers or at the foot of volcanos.

## Informed decisionmaking

Scientific questions, observations and data, relevant theories and methodological tools help scientists to derive new knowledge about natural hazards and vulnerabilities, to assess disaster risks, and to provide scientific evidence to policymakers. Taking a decision in DRR however always requires not only convincing evidence but also a set of options for actions (e.g., how to manage disaster risks; how much to invest in preparedness; what should be the level of awareness to avert panic among the population). Informed decisions with regard to DRR are normally taken across a continuum of urgencies within and beyond national boundaries (e.g., Arimoto et al. 2017; Berkman 2020). Meanwhile, the benefit of preventing losses is not always visible during the term of governmental power. Hence, investments in DRR to avoid losses tend not to be easily accepted in decisionmaking (DM) compared to investments in other urgent issues (such as, developments of new technologies and modern infrastructure or environmental preservation) to gain immediate positive benefits for the society (Ismail-Zadeh and Takeuchi 2007). A cost–benefit analysis provides various options for DM in terms of investments in large and costly DRR projects. But despite the fact that this analysis is a useful tool in optimizing of public fund expenditure, specific options related to non-economic values should be considered in DRR cases, as “the values of human lives, psychological hardness, societal unrest, and other non-economic goods are extremely difficult to evaluate” (Ismail-Zadeh and Takeuchi 2007).

Although scientific knowledge about local and regional NHs is available and constantly advancing, and the local V and E have been analyzed in many places, scientists could make much more to help their governments to build back better, wiser, and stronger. Governments of disaster-affected countries should not cloak the inability of their state institutions to manage disaster risks by employing the ideas that “natural” disasters are unavoidable, or CC is a driver of disasters. But instead they should encourage scientists, engineers, and planners to identify local and regional vulnerabilities, to monitor and reduce these vulnerabilities (e.g., by enhancing building codes), and to propose an improved DRR regulatory policy (Paterson, 2003; Ismail-Zadeh and Takeuchi 2007; AghaKouchak et al. 2018). However, “it is not just a matter of building code and its strict law enforcement alone but the cohesive societal formation that makes various component actions meaningful and combined actions effective” (Ismail-Zadeh et al. 2017). Analyzing the 2019 catastrophic floods in Iran, Bozorg‑Haddad et al. (2021) concluded that monitoring extreme hydrological hazards is not enough to reduce associated disasters, and it should be complemented by a policy of managing and adapting to such events.

In addition, policymakers should promote forensic investigations of disasters happened in order to penetrate deeply into their fundamental causes and to understand why and how NHs turn to become disasters (Ismail-Zadeh 2020b). According to Burton (2010), the aim of such investigations should not be to hunt “witches”, as anyway responsibility for disaster losses is widely spread over institutions and over place and time. Instead the investigations should provide the knowledge of root causes of the disasters to prevent or significantly mitigate similar events in future.

## Conclusion

Principal drivers of disasters are physical and social Vs and E, and not NHs and CC. NH events trigger disasters, and CC is likely to intensify their occurrences, but only where and when the disaster drivers are present. Science has always been at the forefront of research into complex problems related to NHs, CC, V, E, disasters, and associated risks, and scientists have always provided reliable information and compelling evidence of disaster risks to policymakers. However, the COVID-19 pandemic and other disasters associated with NHs (e.g., the 2021 West European floods) showed again that states were not well prepared to tackle with the biological (SARS-CoV2 disease) and other NH extremes, despite the scientific knowledge about the NHs, associated risks, economic and financial consequences of disasters were available for decades.

Disaster science should generate new knowledge in a co-designed and co-productive manner to be delivered to decisionmakers in the form of concise messages and various options for actions to be usable, useful, and used (Boaz and Hayden 2002; Ismail-Zadeh et al. 2017). This knowledge should not be biased by local conditions or by declarations to please policymakers. No political or government actions in reducing disaster risks can be productive without the use of true scientific knowledge, preventive disaster measures, preparedness, and public awareness. Science-based DRR efforts through integrated research and risk assessments (Cutter et al. 2015), and the knowledge exchange using disaster science diplomacy efforts (Kontar et al. 2018, 2021) would help for informed DM to prevent or at least significantly mitigate disasters.

## Data Availability

In accordance with the COPE guidelines on text recycling, the author declares the use in this commentary article of some materials from a few earlier published papers, where he is a principal author. All materials are properly referred.

## Footnotes

1 The policy and practice of reforming the economic and political system (in the former Soviet Union).
